# Loss of the Ash2l subunit of histone H3K4 methyltransferase complexes reduces chromatin accessibility at promoters

**DOI:** 10.1038/s41598-022-25881-0

**Published:** 2022-12-13

**Authors:** Mirna Barsoum, Alexander T. Stenzel, Agnieszka Bochyńska, Chao-Chung Kuo, Alexander Tsompanidis, Roksaneh Sayadi-Boroujeni, Philip Bussmann, Juliane Lüscher-Firzlaff, Ivan G. Costa, Bernhard Lüscher

**Affiliations:** 1 Institute of Biochemistry and Molecular Biology, Faculty of Medicine, RWTH Aachen University, Pauwelsstrasse 30, 52074 Aachen, Germany; 2 Institute for Computational Genomics, Faculty of Medicine, RWTH Aachen University, Pauwelsstrasse 30, 52074 Aachen, Germany; 3 Interdisciplinary Center for Clinical Research (IZKF), Faculty of Medicine, RWTH Aachen University, Pauwelsstrasse 30, 52074 Aachen, Germany

**Keywords:** Molecular biology, Chromatin, Epigenetics, Post-translational modifications, Transcription

## Abstract

Changes in gene expression programs are intimately linked to cell fate decisions. Post-translational modifications of core histones contribute to control gene expression. Methylation of lysine 4 of histone H3 (H3K4) correlates with active promoters and gene transcription. This modification is catalyzed by KMT2 methyltransferases, which require interaction with 4 core subunits, WDR5, RBBP5, ASH2L and DPY30, for catalytic activity. Ash2l is necessary for organismal development and for tissue homeostasis. In mouse embryo fibroblasts (MEFs), Ash2l loss results in gene repression, provoking a senescence phenotype. We now find that upon knockout of *Ash2l* both H3K4 mono- and tri-methylation (H3K4me1 and me3, respectively) were deregulated. In particular, loss of H3K4me3 at promoters correlated with gene repression, especially at CpG island promoters. Ash2l loss resulted in increased loading of histone H3 and reduced chromatin accessibility at promoters, accompanied by an increase of repressing and a decrease of activating histone marks. Moreover, we observed altered binding of CTCF upon Ash2l loss. Lost and gained binding was noticed at promoter-associated and intergenic sites, respectively. Thus, Ash2l loss and reduction of H3K4me3 correlate with altered chromatin accessibility and transcription factor binding. These findings contribute to a more detailed understanding of mechanistic consequences of H3K4me3 loss and associated repression of gene transcription and thus of the observed cellular consequences.

## Introduction

Cells need to respond to endogenous and exogenous signals to modify and adapt their activities to support tissue and organismal functions. The integration of such signals involves complex changes in gene expression programs that can be short-term or long-lasting as for example in response to stress or during differentiation processes, respectively. Central to executing these regulatory programs are sequence-specific transcription factors (sTFs), which exert their effect on chromatin and ultimately on polymerase activity by recruiting transcriptional cofactors^1–3^. These shape chromatin by controlling the density and composition of nucleosomes, modulating the functions of core histones by post-translational modifications (PTMs), and influencing higher-order chromatin organization. Together, these activities allow a multitude of different functional states, which are only incompletely understood. The methylation of histone H3 at lysine 4 (H3K4) has been extensively studied. Trimethylation (H3K4me3) is typically found near transcriptional start sites (TSS) and correlates with accessible promoters^4–7^. Together with H3K27 acetylation (H3K27ac), H3K4me3 marks active promoters, while in combination with H3K27me3, referred to as bivalent chromatin, promoters are poised^8,9^. H3K4me1 is located at enhancers but is also found in promoter regions, where it appears to have a repressive function^10–12^.

Methylation of H3K4 is catalyzed by complexes that contain group 2 lysine methyltransferases (KMT2). These enzymes function in protein complexes referred to as COMPASS (complex of proteins associated with Set1)^4–6^. Six KMT2 enzymes in mammals (MLL1-4 and SET1A and B) associate with the WRAD core complex composed of WDR5, RBBP5, ASH2L and DPY30, as well as other subunits that are complex specific. KMT2 complexes are functionally important, for example, all subunits that have been evaluated in knock-out studies are essential^5,13^. In particular, Ash2l is necessary for organismal development, tissue homeostasis, and cell proliferation and differentiation^14–18^. Moreover, increasing evidence points to critical roles of KMT2 complexes in various diseases^13,19^. For instance, the core subunit Ash2l, which interacts with the oncoprotein c-MYC^20^, has been linked to cancer^21–24^.

The recruitment of KMT2 complexes to chromatin is only partially understood. Several mechanisms have been suggested to be relevant, including the interaction of KMT2 complexes with DNA sequence motifs and structural DNA elements, and the binding to transcription factors and histone marks^25^. For example, binding to GC-rich sequences, including CpG islands (CGIs), has been observed^6^. CFP1, an essential subunit of SET1A/B complexes, binds to non-methylated CpG-rich DNA^26–28^. Some KMT2 complexes, containing SET1A/B, associate with the C-terminal domain of RNA-polymerase II, thereby stimulating the recruitment of these complexes to transcribed genes^29,30^. The function of H3K4me3 at promoters is debated. It has been reported that H3K4me3 is read by the plant homeodomain (PHD) finger of TAF3, a subunit of the general transcription factor complex TFIID. This led to the suggestion that H3K4me3 helps recruit the RNA-polymerase II complex^31–33^. This is supported by the finding that promoting H3K4me3 using a dCas9 system was sufficient to induce gene expression^34^. Moreover, broad and strong H3K4me3 levels at promoters are linked to high transcription^35,36^. Importantly, promoter-associated H3K4me3 can also serve as memory for gene expression^37^. Others find that H3K4me3 might serve as a mark at promoters of transcribed genes, only acquired once transcription has started^38^. However, in Xenopus blocking transcription using α-amanitin has little effect on H3K4me3^39^. In yeast, reduction in H3K4me3 has rather small effects on gene transcription. It was noted that gene silencing was most affected and not gene activation as expected^3,40–45^. Yeast Set1 interacts with active RNA Pol II-dependent on Ser5 phosphorylation of the C-terminal domain (CTD) and thus modifies the 5’-end of transcribed regions^46^. In Drosophila, replacing all histone H3 versions with H3K4R mutants, which cannot be methylated by KMT2 complexes, reduced cell proliferation but did not affect development^47^. Together, these findings suggest roles for H3K4me3 in both activating and maintaining gene transcription.

The knockout of *Ash2l* and of *Dpy30* in murine tissues reduces overall methylation of H3K4, which correlates with altered gene expression^15,18,48^. Using mouse embryo fibroblasts (MEFs), the loss of Ash2l results in broad repression of gene transcription^18^. Phenotypically, the cells stop proliferating and induce a senescence program characterized by a conserved set of downregulated genes. We have now expanded on this work and analyzed H3K4 methylation using chromatin immunoprecipitation combined with next generation sequencing (ChIP-seq). Ash2l loss reduced H3K4me3 at promoters, which was particularly obvious at CGI promoters. Moreover, a general reduced accessibility of chromatin was observed, particularly at promoters, correlating with loss of H3K4me3. The in silico studies of altered transcription factor binding sites of our ATAC-seq data indicated that the transcriptional regulator and chromatin organizer CCCTC-binding factor (CTCF^49–51^) was one of the factors possessing increased DNA binding. Indeed, ChIP-seq experiments supported this notion as CTCF binding increased in intra- and intergenic regions but decreased at promoters. Together, these findings suggest that the loss of Ash2l affects chromatin compaction and that H3K4me3 is involved in maintaining an open chromatin state at promoters.

## Materials and methods

### Immortalized embryonic fibroblasts

Primary mouse fibroblasts were obtained from d13 embryos, immortalized, cultured and manipulated as described before^18^. Immortalized MEF cells with the *Ash2l*^fl/fl^/*Cre-ER* genotype (KO cells) were treated with 5 nM 4-hydroxytamoxifen (+ HOT) (Tocris, 3412) or with vehicle (-HOT, 100% ethanol) to induce recombination of exon 4. This results in loss of protein expression^18^.

### RNA-seq

RNA was obtained from KO cells treated with and without HOT (for 5 days) and sequenced as reported previously. The results were deposited in the Gene Expression Omnibus under the accession number GSE165458^18^. These data were used for the bioinformatic studies described here.

### Chromatin immunoprecipitation (ChIP)-qPCR and ChIP-seq

The antibodies that were used are listed in Table 1. The ChIP experiments were performed using the OneDay ChIP Kit from Diagenode (C01010080) according to the manufacturer’s instructions.

**Table 1:** Antibodies.

Antigen	Species	Company/Cat no	Clonality	RRID number
Primary antibodies ChIP grade				
H3K4me3	Rabbit	Abcam, ab8580	Polyclonal	AB_306649
H3K4me2	Rabbit	Abcam, ab7766	Polyclonal	AB_2560996
H3K4me1	Rabbit	Abcam, ab8895	Polyclonal	AB_306847
H3K9me3	Rabbit	Abcam, ab8898	Polyclonal	AB_306848
H3K9ac	Rabbit	Abcam, ab4441	Polyclonal	AB_2118292
H3K27me3	Mouse	Abcam, ab6002	Monoclonal	AB_305237
H3K27ac	Rabbit	Abcam, ab4729	Polyclonal	AB_2118291
H3K79me3	Rabbit	Abcam, ab2621	Polyclonal	AB_303215
H4K20me2	Rabbit	Abcam, ab9052	Polyclonal	AB_1951942
H3	Rabbit	Abcam, ab1791	Polyclonal	AB_302613
CTCF	Rabbit	Abcam, ab 70303	Polyclonal	AB_1209546
IgG	Rabbit	Diagenode, C01010080	Polyclonal	AB_2722553

Detailed description of ChIP-qPCR experiments with IPs against histone and their respective marks can be found elsewhere^18^. In brief, immunoprecipitations (IPs) were performed with 2 μg of specific antibodies recognizing histone H3 or distinct histone marks. For CTCF 5 µg of a specific antibody was used. The IgG controls were carried out with respective amounts. For IPs 10–100 µg sheared chromatin with a mean size of 500 bp was used. For ChIP-qPCR undiluted 2 μl IP samples and diluted 2 μl input samples were applied in duplicates. A SYBR Green reaction mix (QuantiNova, Qiagen 208054) was employed for the quantitative PCR (qPCR) analyses in a RotorGene 6000 cycler (Corbett/Qiagen). Results were calculated by determining percent input of IPs considering dilution factors. For histone marks a further normalization was performed to percent input obtained with the H3 antibody. The PCR reactions were carried out with an initial step at 95 °C for 2 min, followed by 40 cycles at 95 °C for 10 s, 60 °C for 10 s, and 72 °C for 5 s and a melting curve analysis. One exception was the primer pair CTCF Chr 11 (Table 2). Here an alternative program (95 °C for 2 min, 40 cycle 95 °C for 10 s, 60 °C for 15 s and extension at 72 °C for 20 s) was used, to provide an efficiency > 95%, as for all other primer pairs.

**Table 2:** Oligonucleotides.

Primer name	Sequence (5ʹ–3ʹ)	Function
CHIP-qPCR primers		
Cdh3_ChIP_for	GAGCCACGGGGTACCTTTC	Changes in H3K4 methylation as well as other histone marks accumulated at promoters
Cdh3_ChIP_rev	AGGCACTCTCGAAGCCC	
Flywch2_ChIP_for	CCCGGTATGGTCTACTGACG	
Flywch2_ChIP_rev	AGACCAGAAGAGGGCGTCTA	
Hsp90b1_ChIP_for	GCAACATTTTGGGCACTGGA	
Hsp90b1_ChIP_rev	CTGCAAGTTAGTGGGGCAGA	
Olig1_ChIP_for	GCAAACAAGTCCTGGCCATC	
Olig1_ChIP_rev	AGTGCGCAGTTCAGTCGTTA	
Olfr456_ChIP_for	CTTCAAACCCCCTTTTGGAGC	
Olfr456_ChIP_rev	GCCTTGGGTTCATCACCACT	
Cdh17_ChIP_for	ATCCCTTGAGGCCAAGTGTG	
Cdh17_ChIP_rev	ACTGTGTCCTCCCCAAAAGC	
Atp9a_ChIP_for	GAATTGAGTAGAGCCTCCGAAC	
Atp9a_ChIP_rev	GGTATCAGTGTAGGAAGGAGAGA	
Rab27a_ChIP_for	GAAGAGAAATAGGTCTGCCCATC	
Rab27a_ChIP rev	CTGAAAGCAGCAGGACTCTAAA	
Mapk12_ChIP_for	GGGCTTTAGACTCACGTTCTC	
Mapk12_ChIP rev	GGTGGCCATCAAGAAGTTGTA	
CTCF_Chr8_gained_for	TCTGAGCATCTCTGGTATGAGG	Validation of ChIP-seq-experiments
CTCF_Chr8_gained_rev	AGACAGAAGGGACAGGAACA	
CTCF_Chr12_gained_for	AATGCTGGCTCTTCAGTACC	
CTCF_Chr12_gained_rev	CGTAGAGAGGGATATTGTCTTCA	
CTCF_Chr2_lost_for	TGCGGCTTTGGAAGATCA	
CTCF_Chr2_lost_rev	GCTCTCAGGCAGGGAATAAA	
CTCF_Chr7_lost_for	TCCATCCCTGGTACTGTAAA	
CTCF_Chr7_lost_rev	AAAGAGTGCCCATTCCAAG	
CTCF_Chr19_lost_for	CAGGAAGCGATCAGGAAAGT	
CTCF_Chr19_lost_rev	GCAGGGCTCCTCTAATCTTC	
CTCF_Chr11_stabe_for	GGAAGTGGTGAGTTAGTTCC	
CTCF_Chr11_stable_rev	CACTGCCTGTAAAGATGCAG	
CTCF_Chr5_stabe_for	ACATCCCTGAGCAGAGACAA	
CTCF_Chr5_stable_rev	GCTTTCCCTTCCTTCCATCTTG	

For ChIP-seq experiments, 100 μg chromatin was used per IP, with 10 μg chromatin retained as input control. After immunoprecipitation, as described in the manual of the used Diagenode kit, the complexes were washed in 1 × ChIP buffer, taken up in 200 μl TE (10 mM TrisHCl pH 8.0, 1 mM EDTA) and incubated with 1 μl RNase A (10 mg/ml) at 37 °C for 30 min. After pelleting, the beads were resuspended in 150 μl EB buffer (20 mM TrisHCl pH 7.5, 5 mM EDTA, 50 mM NaCl, 1% SDS) and incubated with 1 μl proteinase K (50 µg/ml) for 2 h at 68 °C. The beads were centrifuged, and the supernatant was transferred to a DNA low binding reaction tube. The beads were resuspended again in 100 μl EB and incubated for additional 5 min at 68 °C. The beads were pelleted, and the two supernatants were pooled. Input DNA was purified by precipitation with five volumes 100% ethanol, incubated for 10 min on ice and centrifuged for 10 min at 10,000×*g*. The supernatant was removed carefully, the DNA pellet dried at room temperature and resuspended in 200 μl EB buffer and treated with RNAse A and proteinase K. Further purification of IP and input DNA was performed using the QIAquick PCR purification Kit (Qiagen, 28106). DNA concentrations in IP and input samples were measured using a Qubit fluorometer (Thermo Fisher Scientific) according to the manufacturer’s instructions. The libraries were prepared using the Next Ultra II DNA library prep kit for Illumina (New England BioLabs, E7645S) and the sequencing was performed as single reads for 75 cycles with a NextSeq 500/550 High Output kit v2.5 (Illumina, 2004906) according to the manufacturer’s recommendations. Multiplex single end sequencing was performed at the EMBL Genomic Core Facility in Heidelberg and at the Genomics Facility of the Interdisciplinary Center for Clinical Research (IZKF) Aachen of the Faculty of Medicine at RWTH Aachen University.

### Assay for transposase-accessible chromatin using sequencing (ATAC-seq)

The basic protocol was performed as described^52^, with adaptions^53^. In brief, crude nuclear extracts were prepared by adding 50 µl of ice-cold ATAC-RSB (10 mM Tris–HCl pH 7.4, 10 mM NaCl, 3 mM MgCl_2_) supplemented with 0.1% NP40, 0.1% Tween-20, 0.01% Digitonin to 5 × 10^4^ cells. Samples were prepared in two technical replicates. Cells were incubated for 3 min on ice. Cooled 1 ml Wash buffer (ATAC-RSB + 0.1% Tween-20) was added, tubes inverted and centrifuged at 500 × g for 10 min at 4 °C. With the pellets of the crude nuclear extracts, a transposition procedure was performed. 50 µl Transposition mix (1xTD buffer, Tagment DNA Enzyme TDE1, both part of the Nextera Rapid Capture Exome Kit #FC140-1000 from Illumina, PBS, 0.1% Tween-20, 0.01% Digitonin) was added to the nuclei and incubated for 1 h at 37 °C with shaking. Purification of transposed DNA was carried out with the Qiagen MinElute PCR Purification Kit (#28004).

Transposed DNA was barcoded using i5/i7 primers of the Nextera Rapid Capture Custom Enrichment Kit (FC-140-1007 by Illumina) and NEBNext Ultra II Q5 Master Mix (#M0544) from New England Labs. Amplification with barcoding primers was done for initial 5 cycles. After this, an aliquot of PCR product was taken and supplemented with SYBR Green in DMSO (SYBR Gold Nucleic Acid Gel Stain #S11494, Thermo Fisher) together with the matching set of primers per sample and fresh Q5 polymerase. Another 20 cycles were performed with this aliquot and the amplification was monitored in a real-time application (RotorGene 6000, Qiagen) to assess the progress of the library preparation after the initial 5 cycles in the thermocycler. For both + HOT treated and one -HOT treated sample 4 additional and for the second -HOT replicate 3 additional amplification cycles were needed to perform to extend the initial 5 cycles of preparation. After library preparation was done, samples were purified with the Qiagen MinElute PCR Purification Kit (#28004).

The libraries were sequenced as paired-end reads for 75 cycles with a NextSeq 500/550 High Output kit v2.5 (Illumina, 20024906) according to the manufacturer’s recommendations. Sequencing and de-multiplexing were done by the Genomics Facility of the Faculty of Medicine at RWTH Aachen University.

### Quantification and statistical analysis

Error bars represent standard deviation (SD) of the mean, unless otherwise indicated. Statistical significance was evaluated by multiple t-test using GraphPadPrism software, unless otherwise indicated.

### Bioinformatics

For both ChIP-seq and ATAC-seq, sequences were trimmed using Trim_Galore (https://www.bioinformatics.babraham.ac.uk/projects/trim_galore/). They were then aligned against the reference genome (mm9) using BWA^54^. We used the view, sort and index functions of SAMtools to convert the Sam to Bam files and sort and index the mapped reads^55^. The complexity of the genomic sequencing library was checked using preseq (https://github.com/smithlabcode/preseq). The duplicates were marked using Picard MarkDuplicates (https://github.com/broadinstitute/picard/blob/master/src/main/java/picard/sam/markduplicates/MarkDuplicates.java). We filtered the un-mapped reads, PCR-duplicates, blacklist region as defined by ENCODE and, in case of paired-end sequencing (ATAC-seq), the un-paired reads using the view function of SAMtools^55^. We checked the enrichment and quality of ChIP-seq (H3K4me3, H3K4me1 and CTCF) using the plotFingerprint function of DeepTools^56^ and Rscript run_spp.R in the phantumpeakqualtools package (https://github.com/crazyhottommy/phantompeakqualtools/blob/master/run_spp.R).

The quality of the ATAC-seq was evaluated by checking the insert size distribution using the CollectMultipleMetrics function of Picard (https://github.com/broadinstitute/picard/blob/master/src/main/java/picard/analysis/CollectMultipleMetrics.java). MultiQC was used to merge all reports from the same experiment^57^. Narrow peaks (ChIP-seq (H3K4me3), ATAC-seq and ChIP-seq (CTCF)) and Broad peaks (ChIP-seq (H3K4me1)) were called using Macs2^58^. In ChIP-seq (CTCF) experiments, motif-analysis of CTCF consensus sites at topologically associating domain (TAD) boundaries was performed using the FIMO (Find Individual Motif Occurrences) program from the MEME suite^59^. TAD boundaries were obtained from published data^60^ (bed-files at http://chromosome.sdsc.edu/mouse/hi-c/index.html). The overlap was examined by considering a resolution of ± 20 kb regarding the published Hi-C data. Intersecting between different experiments was done using BEDTools^61^. The computeMatrix and the plotheatmap functions of DeepTools were used to calculates the scores per genome region in each sample and then ploted the heatmaps^56^. These were normalized using CPM (count per Million) in ChIP-seq (H3K4me1 and me3) and ATAC-seq (the merged files of the two technical replicates). For ChIP-seq (CTCF), the BigWig tracks were normalized using the scale factors obtained by Deseq2.

For both ChIP-seq (CTCF) and ATAC-seq, we used DEseq2 to normalize the raw counts in the two technical replicates of each condition and to perform differential analysis between -HOT and + HOT^62^. For ChIP-seq (H3K4me1 and me3), the counts were normalized to the lowest coverage and the logFC was calculated manually for each biological replicate (KO1 and KO2). Individual logFC threshold to call gained and lost peaks in + HOT compared to -HOT for each of the above-mentioned sequencing experiments was determined after visualization in IGV (Integrative Genomics Viewer, https://software.broadinstitute.org/software/igv/). The called gained and lost peaks were annotated using Homer (http://homer.ucsd.edu/homer/ngs/annotation.html). The information about the distance to the nearest promoter provided by Homer after the annotation was used to annotate the peaks as promoters (± 3000 bp of the TSS). We also grouped the counts of the H3K4me3 binding sites at promoters by their A value (log(counts in -HOT and + HOT)) in KO1 and KO2, which is estimated from the MA plots (Supplementary Fig. S1b) and IGV as follows: higher than 14 (high), between 14 and 10 (medium), and lower than 10 (low) for KO1. Higher than 19 (high), between 19 and 12 (medium) and lower than 12 (low) for KO2. In ChIP-seq (H3K4me1 and me3), motif enrichment analysis and histone line plots were performed with the Regulatory Genomics Toolbox (RGT; www.regulatory-genomics.org) based on promoter sequences 1000 bp upstream and 100 bp downstream of the TSS. Motifs were obtained from Jaspar version 2020^63^. Promoter sequences 1000 bp upstream to 100 bp downstream the TSS were enriched for TATA box and GC-rich motifs provided by the Eukaryotic promoter database^64^. The IGV genome browser was used to produce screenshots of selected genomic locations. The enhancers’ genomic locations were obtained from the EnhancerAtlas 2.0 (http://www.enhanceratlas.org/indexv2.php)^65^. Coordinates of CpG islands were obtained from UCSC (https://hgdownload.soe.ucsc.edu/goldenPath/mm9/database/cpgIslandExt.txt.gz).

In ATAC-seq, the two technical replicates were merged before the transcription factor (TF)-footprinting analysis using Picard MergeSamFiles (https://github.com/broadinstitute/picard/blob/master/src/main/java/picard/sam/MergeSamFiles.java). TF-footprinting and thereafter the TF-differential analysis were performed using RGT-HINT^53^. Part of the codes used in this manuscript were modified from nf-core (https://nf-co.re/chipseq/ and https://nf-co.re/atacseq).

All sequencing data are available in NCBI’s Gene Expression Omnibus (GEO)^66^ as SuperSeries under accession number GSE205233 (https://www.ncbi.nlm.nih.gov/geo/query/acc.cgi?acc=GSE205233. This SuperSeries is composed of the following sub-series: 1. Accession number GSE205232 for ChIP-seq (H3K4me1, H3K4me3). 2. Accession number GSE205230 for ATAC-seq. 3. Accession number GSE205231 for ChIP-seq (CTCF).

## Results and discussion

### Altered H3K4 methylation at promoters upon loss of Ash2l

**Figure 1:**
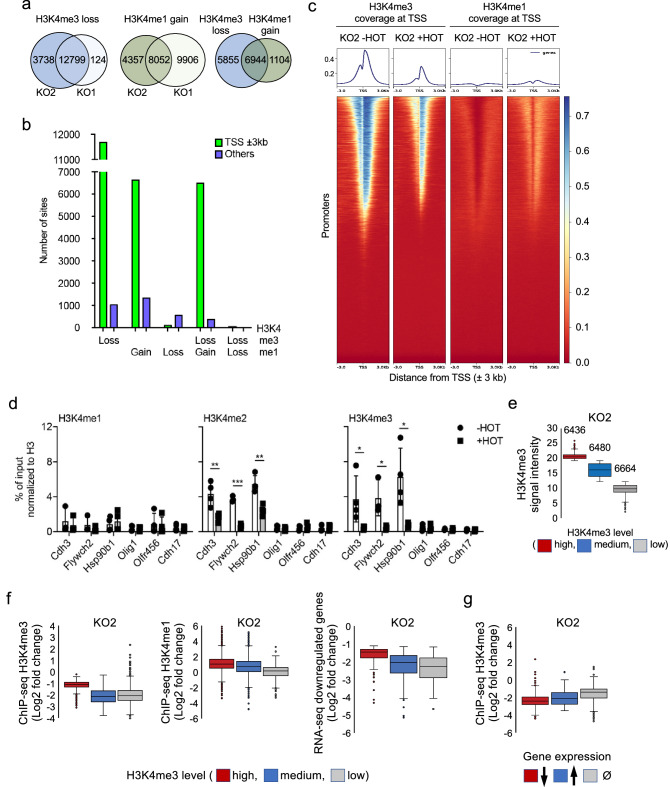
Deregulated H3K4me3 and me1 at promoters in response to Ash2l loss. (**a**) ChIP-seq analysis using antibodies against H3K4me3 and me1 with wild type (WT) and *Ash2l*^*fl/fl*^ (KO) immortalized fibroblasts (iMEF1 and 2) incubated ± HOT for 5 days. The total number of sites that gain or lose signals are indicated (logFC > 0.58 and signals of > 20 reads). (**b**) ChIP-seq data as in panel A. Gained and lost H3K4me3 and me1 signals at promoters were analyzed (± 3000 bp; logFC > 0.58 and signals of > 20 reads). No gain of H3K4me3 was observed. Promoters with a loss of both marks were rare. (**c**) Heatmaps generated using DeepTools showing H3K4me3 and me1 normalized signals (normalized using counts per Million (CPM)) obtained from KO2 cells in the presence and absence of Ash2l (± HOT) at all annotated transcripts in mm9. (**d**) ChIP-qPCR experiments of KO2 cells treated ± HOT for 5 days. Mean values ± SD of 4 independent experiments measured in duplicates are displayed (* < 0.05, ** < 0.01, *** < 0.001). (**e**) Promoters were classified according to their H3K4me3 levels. Three categories were generated, the numbers of promoters in each category are indicated. (**f**) Promoters (± 3000 bp of TSS) with high, medium and low H3K4me3 signals were compared regarding alterations in H3K4me1 and H3K4me3 and changes in expression of the corresponding genes. (**g**) Genes that are up- or downregulated or did not change in expression were compared to changes in H3K4me3 at their promoters.

The loss of Ash2l in both hematopoietic and MEF cells results in inhibition of proliferation. At the molecular level, a reduction of H3K4 methylation and altered gene expression was observed^15,18^. In MEF cells this correlates with the induction of senescence. To further evaluate H3K4 methylation, we performed ChIP-seq of 2 pairs of Ash2l KO and WT immortalized MEF cells (i.e. iMEF1 and 2, for details see^18^) at day 5 after 4-hydroxytamoxifen (HOT) treatment, resulting in deletion of exon 4 of *Ash2l* and loss of Ash2l protein. Genome-wide, 22,344 and 122,781 H3K4me3 and me1 modified regions, respectively, were identified. A large number of H3K4me3 marked chromatin sites (12,799 common for KO1 and KO2 cells) showed loss of signal upon HOT treatment of KO cells (log2FC > 0.58; signals of > 20 reads) (Fig. 1a and Supplementary Table S1; also available in GEO under accession number GSE205232). The vast majority of these sites were associated with promoters (± 3000 bp of the transcriptional start site (TSS)). Roughly a third of the 37,205 promoters analyzed showed loss of H3K4me3 (Fig. 1b,c and Supplementary Fig. S1a), consistent with the decrease in global H3K4me3^18^. The decrease in H3K4me3 was particularly obvious in regions with intermediate levels of this modification as depicted in MA plots (Supplementary Fig. S1b).

H3K4me1 marks enhancers^11,12^. Of the large number of H3K4me1 modified regions, fewer than 600 lost signals (Fig. 1b), consistent with the small decrease in global H3K4me1^18^. One possibility is that in the absence of Ash2l KMT2 enzymes might possess mono-methyltransferase activity. In vitro studies suggest that at least some KMT2 complexes with WDR5 and RBBP5 mono- and di-methylate H3K4, while the addition of Ash2l promotes tri-methylation and stimulates overall activity^67–69^. However, our Rbbp5 immunoprecipitates did not contain methyltransferase activity in the absence of Ash2l^18^. Alternatively, H3K4me1 might be sufficiently stable during the course of the experiment, preventing loss of signal. We noticed that some H3K4me1 marked sites gained signals (Fig. 1a; 8052 common for KO1 and KO2; log2FC > 0.58; signals of > 20 reads). Most of these sites were accompanied by a decrease in H3K4me3 and are linked to promoters (Fig. 1b,c and Supplementary Fig. S1a–c).

The changes in H3K4 methylation, as described above, are documented in the displayed IGV browser tracks at the *Cdh3* locus, which lost H3K4me3 and gained H3K4me1 in its promoter region (Supplementary Fig. S1c). These effects were validated for *Cdh3* and the promoters of several additional genes. *Cdh3* and *Flywch2* are downregulated in response to Ash2l loss, while the expression of *Hsp90b1* was unchanged in the RNA-seq experiments (summarized below in Supplementary Fig. S1d)^18^. All three lost H3K4me3 and me2 in their promoter regions (Fig. 1d). However, the increase in H3K4me1 was less obvious. *Olig1*, *Olfr456* and *Cdh17* are genes that were minimally or low expressed in WT and untreated KO cells^18^, and showed no H3K4me3 in their promoter regions in the ChIP-seq data set in KO1 and KO2 iMEFs (Supplementary Table S1). This was corroborated in ChIP-qPCR experiments, demonstrating low levels of all three H3K4 methylation states (Fig. 1d, summarized in Supplementary Fig. S1d). Of note, in the HOT treated KO1 and KO2 cells the expression of *Olfr456* and *Cdh17* was upregulated, the latter only in RT-qPCR measurements, but not of *Olig1*^18^*.* Thus, these findings support the concept that H3K4me3 correlates with gene expression and that H3K4 methylation at promoters is broadly affected in response to loss of Ash2l. In contrast, the H3K4me1 pattern was remarkably stable with an increase in regions that carried H3K4me3 modifications suggesting that the loss of tri- and di-methylation resulted in an increase in mono-methylation.

### Gene repression correlates with loss of H3K4me3

We evaluated the correlation between changes in the H3K4 methylation patterns and gene expression. Promoters were grouped according to low, medium and high H3K4me3 signals (see material and methods section for details; Fig. 1e and Supplementary Fig. S1e). We observed that the fold reduction of H3K4me3 in the high group was the lowest (Fig. 1f, left panel, and Supplementary Fig. S1f). Despite this, these promoters revealed the highest increase in H3K4me1 (Fig. 1f, middle panel, and Supplementary Fig. S1f), supporting the suggestion that the loss of both H3K4me3 and me2 resulted in an increase in H3K4me1, particularly at promoters with very high H3K4me3. H3K4me1 may then persist as this modification appears to be rather stable (Fig. 1a,b). Also, the genes associated with H3K4me3^high^ promoters were those with the smallest decrease in expression, while those genes with H3K4me3^medium^ and H3K4me3^low^ promoters were downregulated more strongly (Fig. 1f, right panel, and Supplementary Fig. S1f). One interpretation is that H3K4me3^high^ promoters possess, after 5 days of HOT treatment, still sufficient H3K4me3 for being efficiently transcribed and that a certain H3K4me3 threshold is required to maintain accessibility of promoters and thus allow transcription.

This is consistent with promoters of downregulated genes showing the largest decrease in H3K4me3, while the decrease was smaller for the few upregulated genes (Fig. 1g and Supplementary Fig. S1g). At present, it is unclear whether this increase in RNA is due to enhanced transcription or due to stabilization of the RNA as a consequence of the overall repression of gene transcription and thus some secondary effect. Further evaluation may require a system that allows short-term regulation of Ash2l to acquire the ability to study more direct effects of Ash2l loss.

### GC-rich promoters are sensitive to loss of H3K4me3

**Figure 2:**
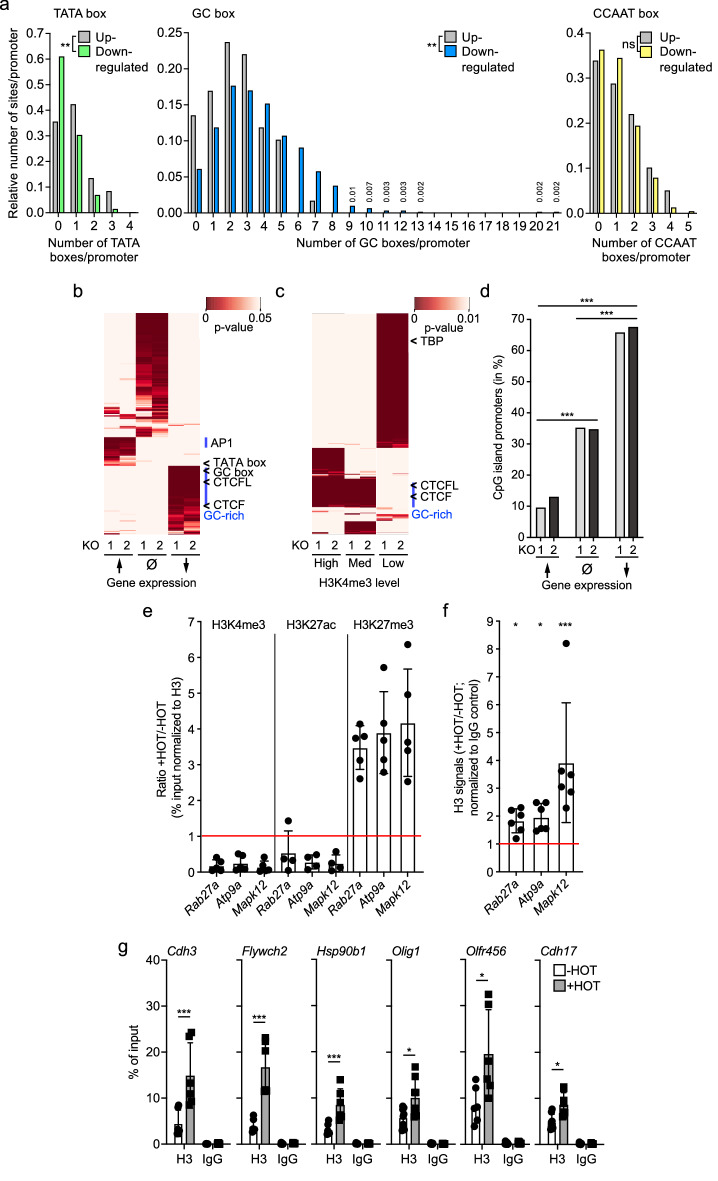
CpG island promoters are preferentially affected upon Ash2l loss. (**a**) The presence of TATA, GC and CCAAT boxes in promoter region of genes (-1000 to + 100 bp) that were up- or downregulated. Kolmogorov–Smirnov test: ** < 0.01; ns, not significant. (**b**) Putative transcription factor binding sites in promoters of up- or downregulated genes and of genes that did not show significant changes in expression (Ø). Indicated are the TATA box and the GC box and areas in which binding sites for AP1 proteins (AP1) and factors that interact with GC-rich sequences (GC) are preferentially found. Moreover, CTCF and CTCFL binding sites are indicated. (**c**) Putative transcription factor binding sites of promoters with high, medium, or low H3K4me3. Indicated sites are as described in panel (**b**). (**d**) Indicated are the percentage of CpG island promoters of genes that are up- or downregulated or unaffected in KO1 and KO2 cells in response to Ash2l loss (upregulated: 176 genes in KO1 and 206 genes in KO2; downregulated: 1118 genes in KO1 and 1600 genes in KO2; not changed: 35,911 genes in KO1 and 35,399 in KO2). Chi-square with Yates correction, two tailed: *** < 0.001. (**e**,**f**) Three genes with promoters characterized by strong CGIs were analyzed for the presence of the indicated histone marks (panel **e**) and H3 occupancy (panel **f**) using ChIP-qPCR of KO2 cells treated ± HOT for 5 days. Mean values ± SD (n = 4–6) are given (* < 0.05, *** < 0.001). (**g**) Histone H3 ChIP-qPCR of promoter regions of the indicated genes in iMEF KO2 cells treated ± HOT for 5 days. Rabbit IgG served as control. Mean values ± SD (n = 6) are given (* < 0.05, ** < 0.01, *** < 0.001).

Two major types of promoters have been classified according to either a focused or a dispersed TSS^70,71^. The former is typically characterized by the presence of a TATA box as a core promoter element. The latter is associated with CpG islands (CGIs) and thus are enriched for GC-rich binding sites. These include the GC box, originally defined as SP1 binding site^72^, and more general sites for SP as well as Krüppel-like factors (KLF)^73–76^. We compared the presence of TATA and GC boxes in promoters of up- and downregulated genes. Downregulated genes were increased for promoters with GC boxes while TATA boxes were reduced (Fig. 2a–c)^64^. As control, CCAAT boxes, which are recognized by TFs such NF-Y and C/EBP^74,77^, were equally distributed between up- and downregulated genes (Fig. 2a). Consistent with these findings was that GC-rich binding sites for SP and KLF transcription factors were also increased in downregulated genes (Fig. 2b and Table 3; full data set in Supplementary Table S2; also available in GEO under accession number GSE205232). For example, 76% and 57% of downregulated genes in KO1 or KO2 cells, respectively, possess Klf4 and SP1 binding sites within their promoter proximal regions supporting the conclusion that GC-rich promoters are preferentially downregulated (Supplementary Table S2). Similarly to SP and KLF sites, CTCF and CTCFL consensus sites were increased, which also have a high GC content (Fig. 2b,c). We note that CTCFL is not expressed in our MEF cells according to the RNA-seq data^18^, consistent with its expression being very low in normal somatic cells^49,51^. Many CTCF and CTCFL binding sites overlap with some marked with H3K4me3 and thus most likely represent promoters^78,79^. Together, GC-rich binding sites were preferentially associated with promoters characterized by high and medium H3K4me3 (Fig. 2c). Additionally, an increase in binding sites for AP1 factors, including JUN and FOS proteins, was observed for upregulated genes (Fig. 2b. Table 3 and Supplementary Table S2). Finally, in support of the association of GC boxes with repressed genes, the majority of downregulated genes are controlled by CGI promoters, while only few CGIs are linked to upregulated genes (Fig. 2d). Together these findings suggest that the consequences of a loss of Ash2l and thus of H3K4 methyltransferase activity, are particularly pronounced at CGI promoters.

**Table 3:** Transcription factor binding sites associated with up- and downregulated genes.

Group	Name	Down in KO1	No change in KO1	Up in KO1	Down in KO2	No change in KO2	Up in KO2
KLF	KLF1-6, 9-12, 16, 15-17	#	ns	ns	#	ns	ns
SP	SP1-4, 8-9	#	ns	ns	#	ns	ns
CTCF, CTCFL	CTCF, CTCFL	#	ns	ns	#	ns	ns
ZNF	ZNF148, ZNF449, ZNF16, ZNF423, ZNF263, ZNF740, ZNF460, ZNF682, ZNF684, ZNF281	#	ns	ns	#	ns	ns
JUN	JUN, JUNB, JUND	ns	ns	#	ns	ns	#
MAF	MAFB	ns	ns	#	ns	ns	#
FOS	FOS, FOS 1	ns	ns	#	ns	ns	#
BATF	BATF	ns	ns	#	ns	ns	#

^#^p < 0.05; ns, not significant (TF binding sites are not significantly enriched).

### Ash2l loss affects promoter associated histone H3 loading and histone marks

H3K4me3 correlates with promoter accessibility and transcription^5,7^. Thus, loss of H3K4me3 may result in less accessible, and thus potentially more compacted chromatin at promoters. We chose the six genes analyzed above (Fig. 1d and Supplementary Fig. S1d). In addition, three genes were selected with strong CGI promoters (*Rab27a*, *Atp9a* and *Mapk12*), which lost H3K4me3 upon *Ash2l* KO (Fig. 2e). The level of histone H3 at promoters was assessed using ChIP-qPCR (for a summary of changes in H3K4me3 and expression upon HOT treatment, see Supplementary Fig. S1d). The H3 ChIP signal in the *Ash2l* KO samples increased at all 9 promoters upon Ash2l loss (Fig. 2f,g). In addition, we observed a decrease of H3K27ac and an increase in H3K27me3 at the majority of the promoters (Fig. 2e and Supplementary Fig. S2). In support for less accessible chromatin, H3K9ac was decreased (Supplementary Fig. S2). Finally, we measured H3K79me2/3, enriched in the transcribed regions of active genes and with functions in the response to DNA damage^80^, and H4K20me2, associated with DNA repair^81^, which were largely unchanged at the evaluated promoters (Supplementary Fig. S2). The impact on modification of H3K27 may relate to observations that KMT2 complexes have been reported to be associated with KDM6/UTX enzymes, which demethylate H3K27, and CBP/p300, which acetylate H3K27^20,82–84^, thus supporting the strong interplay of H3K4 and H3K27 marks^9^. Together, these findings suggest that the loss of Ash2l results in less accessible chromatin at promoters and a shift from activating to repressing chromatin marks, which is particularly evident at CGI promoters.

### Decreased chromatin accessibility upon loss of Ash2l

**Figure 3:**
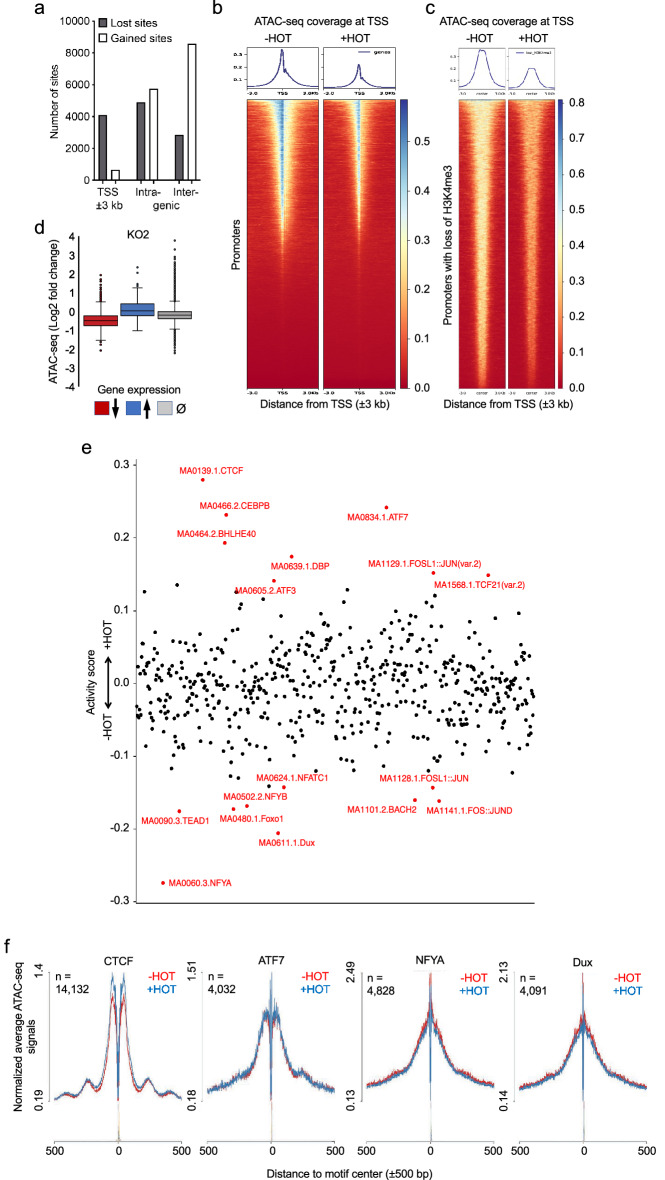
Ash2l loss induces chromatin compaction. (**a**) Lost and gained ATAC sites as defined after differential analysis using Deseq2 (q < 0.05; log2FC > 0.4) were analyzed at promoters (± 3000 bp of TSS), and in intra- and intergenic regions. (**b**) Heatmaps generated using DeepTools showing normalized ATAC-seq signals (normalized using counts per million (CPM)) in the presence and absence of Ash2l (± HOT) at all annotated transcripts in mm9 (± 3000 bp of TSS). (**c**) Heatmaps comparing chromatin accessibility by ATAC of promoters that lost H3K4me3 in the presence or absence of Ash2l (± HOT). (**d**) The promoters of genes that are up- or downregulated or did not change in expression in response to ± HOT treatment were evaluated regarding their accessibility in the ATAC-seq approach. (**e**) Transcription factors (TFs) footprinting and their differential analysis in the ATAC-seq data set were performed using RGT-HINT. In red are TFs with more than 1000 binding sites with altered accessibility (q < 0.05). A summary of these sites is given in Supplementary Table S4. (**f**) Line plots for two TFs showing lost activity (increased binding of CTCF and ATF7) and two TFs with gained activity (decreased binding of NFYA and Dux) upon Ash2l loss in KO2 as defined in panel (**e**).

To further evaluate a possible chromatin compaction upon Ash2l loss, we performed ATAC-seq experiments at day seven of HOT treatment. These revealed the expected pattern of nucleosome-free regions, mono-nucleosomes, di-nucleosomes and larger fragments (Supplementary Fig. S3a). The significantly changed sites upon loss of Ash2l (q < 0.05; log2FC > 0.40), 15,087 sites gained and 11,961 sites lost accessibility, were analyzed regarding their location (Supplementary Table S3; also available in GEO under accession number GSE205230). We compared the accessibility of promoter regions (± 3 kb) to intra- and intergenic regions of the genome. The gained accessibility was preferentially in the intra- and intergenic regions (Fig. 3a). Considering that a 6000 bp region of 37,205 promoters was analyzed, which represents roughly 8.3% of the murine genome, the gained sites were slightly underrepresented at promoters (4.6% of total gained sites when assuming one site/6 kb fragment). Lost accessibility was predominantly near promoters (34.6% of total lost sites). Thus, they were 4.5-fold more abundant than expected, suggesting that promoter regions were preferentially less accessible upon Ash2l loss (Fig. 3a,b). Although it has been argued that the promoters of transcribed compared to silent genes are more accessible, only few studies have provided evidence for a link to H3K4me3. In two distinct experimental systems, murine myogenesis and embryogenesis in Xenopus, H3K4me3 signals correlate with accessibility by ATAC-seq analysis, but because the histone mark was not manipulated functional links were not established^85,86^. Thus, our findings suggest that the loss of H3K4me3 compromises promoter accessibility. We note that the time frame in our experimental system is rather long and only when short term regulation of this histone mark will be achieved, conclusions about potentially direct consequences might become possible.

Altered accessibility was particularly obvious just upstream of the TSS, a region that is typically nucleosome-depleted when genes are transcribed^87–89^. Therefore, we addressed whether an increase in mono-nucleosomes close to the TSS can be detected when a smaller region encompassing ± 600 bp is evaluated (Supplementary Fig. S3b). This revealed that the overall accessibility in this small chromatin window was reduced but we did not observe a significant increase in positioned nucleosomes at or just upstream of the TSS. We then analyzed the promoters of downregulated genes, which might be affected more strongly, however, the effect of Ash2l loss was similar with a decrease of the overall accessibility (Supplementary Fig. S3b). Further comparison of the different data sets documented that chromatin regions with H3K4me3 loss became compacted (Fig. 3c). Finally, chromatin compaction was most prominent at promoters of downregulated genes (Fig. 3d). Together, the increased compaction at promoters upon Ash2l loss was consistent with an increase in H3 signals, and thus likely due to increased nucleosome loading. However, a well-positioned nucleosome just upstream of the TSS^87^, which we expected to result in a distinct pattern of ATAC-seq signals, could not be visualized. Whether this is due to not fully established changes in chromatin organization at the chosen time point and/or due to variability in the position of postulated upstream nucleosomes relative to the TSS, remains to be determined.

To evaluate whether the observed alterations in the accessibility of DNA were associated with distinct DNA motifs, the ATAC fragments were screened for transcription factor (TF) binding sites. We noticed that a few sites were strongly linked to altered accessibility (Fig. 3e). For further analysis, we concentrated on those sites that showed significantly changed activity upon Ash2l loss (p < 0.05) and, in addition, for which at least 1000 binding sites were observed in our ATAC-seq data set. At this stringency, we identified 8 TF binding motifs that gained and 9 that lost occupancy (Fig. 3e and Supplementary Table S4; also available in GEO under accession number GSE205230). Of those TF motifs that significantly gained binding activity, CTCF sites were affected most profoundly. CTCF binds to GC-rich sequences, which are associated with downregulated genes (Fig. 2a–c), and has major functions as transcriptional regulator and in higher-order chromatin organization^49,50^. Alterations of activity were identified for 14,132 sites in the ATAC-seq data set (Fig. 3f and Supplementary Table S4). Overall, higher sequence coverage was observed on both sides of CTCF consensus DNA binding sequences (Fig. 3f). For comparison, increased binding to ATF7 consensus sites, and decreased binding to NFYA and Dux consensus sites are displayed, which showed weakly altered protection compared to CTCF (Fig. 3e,f). Moreover, the analysis of the neighboring regions of the CTCF consensus motif suggested that the positioning of both the − 1 and + 1 nucleosomes was enhanced (Fig. 3f). Well positioned nucleosomes flanking CTCF sites have been noted previously^90–93^. This suggested that the altered accessibility of chromatin was linked to relatively few known TF binding motifs.

### Binding of CTCF to core promoters is reduced upon Ash2l loss

**Figure 4:**
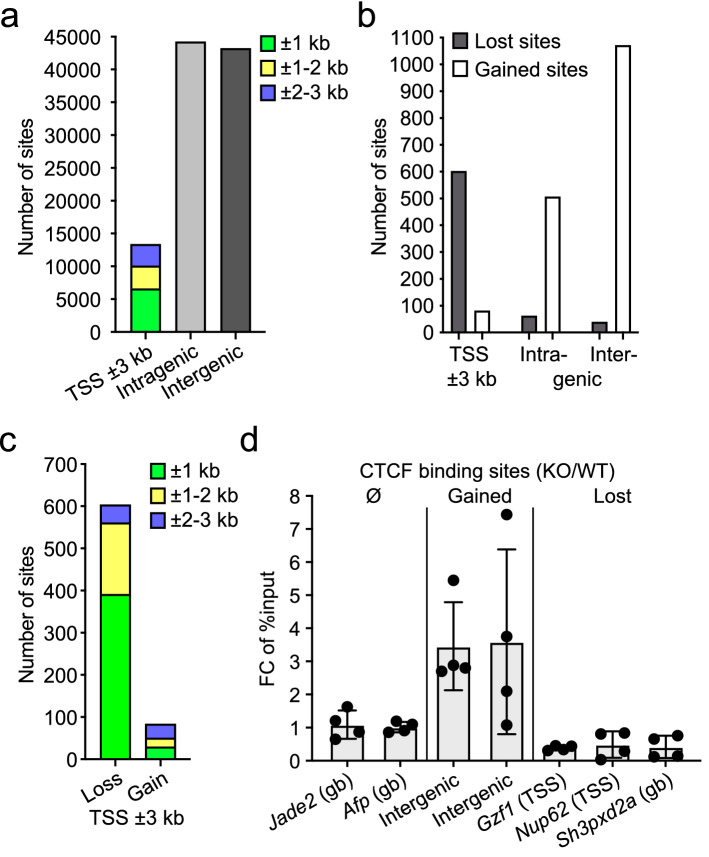
The accessibility of CTCF binding sites is altered in response to Ash2l loss. (**a**) ChIP-seq analysis of KO2 cells (2 replicates) treated for 7 days ± HOT. All detected CTCF binding sites in -HOT and + HOT treated cells were grouped to promoter (TSS ± 3000 bp), intragenic and intergenic regions. (**b**) Lost and gained CTCF binding sites as defined after differential analysis using Deseq2 (q < 0.05; logFC > 1) of the three regions indicated as in panel (**a**). (**c**) Lost and gained CTCF peaks near the TSS. The region is divided into three intervals on both sides of the TSS, until 1 kb, from 1–2 kb and from 2–3 kb. (**d**) CTCF ChIP-qPCR of loci that gained or lost binding or were unaffected. The relevant genes are indicated. The intergenic sites (left) are on chromosome 8 (Chr8:3,955,70-3,956,485) and on 12 (Chr12: 89,947,682-89,948,082) (left and right bar, respectively).

Because of the effects related to CTCF binding site motifs in our ATAC-seq data, we performed CTCF ChIP-seq experiments of control and 7 day HOT treated cells in replicates. We identified a total of 101,513 binding sites (Fig. 4a and Supplementary Table S5; also available in GEO under accession number GSE205231), which is in the same order of magnitude as reported by others. For example, when the CTCF occupancy landscape in 40 different human cell lines was determined, an average of 61,944 sites and a total of 107,295 sites across the different cell lines were detected^94^. Moreover, in murine cells two- to threefold more CTCF sites were noticed when compared to human cells^95^. Of those sites that showed altered binding upon knockout of *Ash2l* (q < 0.05; log2FC > 1), a loss was observed at 719 and a gain at 1682 binding sites (Supplementary Table S5). Of note was that most of the losses were located in promoter regions (TSS ± 3000 bp) (Fig. 4b). When we further subdivided the ± 3000 bp window, we observed that lost binding sites were enriched close to the TSS in the ± 1000 bp window and their numbers decreased with increasing distance to the TSS, consistent with the ATAC-seq data (Fig. 4c). Compared to a statistically distributed change in CTCF binding sites, we observed a 10.2- and 19.6-fold increase in lost CTCF binding sites in the ± 3000 and ± 1000 promoter window, respectively. Thus, the loss of CTCF binding was even more pronounced than the effect on accessibility measured by ATAC-seq (see above). For verification, the differential occupancy of CTCF sites in response to Ash2l loss at different genomic locations, as determined by ChIP-seq, was measured in independent ChIP-qPCR experiments (Fig. 4d and Supplementary Fig. S4a). At 7 distinct loci, 2 unaffected, 2 with increased and 3 with reduced CTCF binding in the ChIP-seq data set, the alterations were reproducible. Our findings are consistent with previous notions that CTCF binding is in competition to a fragile nucleosome close to the TSS^96,97^, and with occupation of promoter-linked CTCFL sites being negatively correlated with H3 loading^79^.

Next, we compared the CTCF binding sites that were gained/lost upon Ash2l depletion with the set of up-/downregulated genes^18^. Although a small number of downregulated genes lost CTCF binding in their promoter regions, the majority of lost CTCF sites were not associated with the promoters of significantly downregulated genes (Supplementary Fig. S4b). This suggested that the loss of CTCF binding at promoters is unlikely to play a major direct role in gene repression upon Ash2l loss. The intersection of CTCF gained peaks and upregulated genes with the other two groups was minimal (Supplementary Fig. S4b). Thus, also the upregulated genes were unlikely to be main targets of CTCF. Furthermore, we compared our CTCF ChIP-seq data set with annotated enhancers in MEF cells^65^. Of note was that at enhancers decreased CTCF binding was observed (Supplementary Fig. S4c). Although the number of significantly altered CTCF binding sites was low, their reorganization may affect clustering of transcriptional regulators, thereby modulating gene expression^98^.

Because CTCF binding sites are associated with topologically associating domain (TAD) boundaries^49^, we compared our CTCF ChIP-seq data set with TAD boundaries that were determined in mouse embryonic stem cells (mESCs)^60^, as no defined positions of annotated TAD boundaries for MEFs were available. Therefore, this comparison has to be interpreted with caution. We found that both gained and lost CTCF peaks were associated with potential TADs in MEF cells (Supplementary Fig. S4d). Of the gained peaks, 13% overlap with TADs, while of the lost peaks 30% are TAD associated. This suggested that higher-order chromatin organization was affected upon Ash2l loss. Considering that 15% of CTCF are residing at TAD boundaries^60,99^, these numbers are compatible with this interpretation. Together, these findings suggest that altered CTCF binding sites are linked to chromatin organization, and thus may affect gene expression indirectly, rather than to regulatory functions proximal to promoters.

### The role of H3K4me3 in reorganizing active CTCF binding sites in *Ash2l*-KO MEF cells

**Figure 5:**
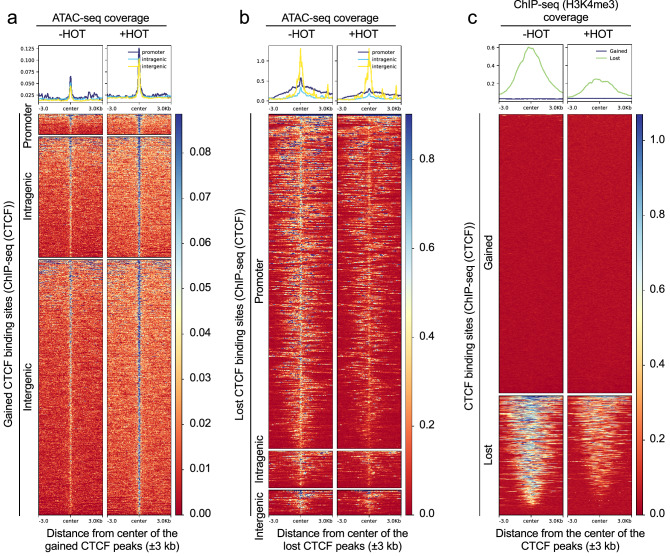
Loss of CTCF peaks at promoters is accompanied by a loss of H3K4me3 and an increased chromatin compaction. (**a**) Heatmaps showing the normalized ATAC-seq signals centered at gained CTCF binding sites (q < 0.05; logFC > 1) grouped to promoters (± 3000 bp of TSS), and intra- and intergenic regions. (**b**) Heatmaps showing the normalized ATAC-seq signals centered at lost CTCF bindings sites as in panel (**a**). (**c**) Heatmaps showing the normalized H3K4me3 signals centered at gained and lost CTCF binding sites (q < 0.05; log2FC > 1; ± 3000 bp).

To further compare the different data sets, we used the 1682 CTCF binding sites that gained binding in response to Ash2l loss in the ChIP-seq experiments and asked how this increased binding affected the neighboring chromatin. We observed increased accessibility around the CTCF binding sites (Fig. 5a). This was found for sites near the promoter (TSS ± 3000 bp) and also for intragenic and intergenic sites. When lost CTCF binding sites were analyzed, reduced accessibility was noted in the promoter regions (Fig. 5b), consistent with the overall decrease in promoter accessibility. Similar tendencies were noted for the lost sites in intragenic regions, but not for intergenic regions, although the number of affected sites was small in both intra- and intergenic regions. Finally, we compared the lost and gained CTCF sites regarding colocalized H3K4me3 signals. As the lost sites are predominantly near promoters (Fig. 4b), we expected a decrease in H3K4me3. Indeed, this was observed (Fig. 5c). In contrast, the gained sites, which are predominantly intra- and intergenic, showed very low H3K4me3 signal that did not change upon Ash2l loss (Fig. 5c). These findings suggest that CTCF dissociates after Ash2l and H3K4me3 loss from core promoter regions and may redistribute to more accessible intergenic sites. Whether this is a direct consequence of H3K4me3 depletion and chromatin compaction needs to be further investigated.

## Conclusions

Our findings suggest that Ash2l loss and concomitant reduction in H3K4 methylation results in chromatin compaction. This is exemplified by the increased histone H3 ChIP-qPCR signals at selective promoters and the overall decreased accessibility of promoters in the ATAC-seq experiments. This is consistent with the observation that active TSSs are preferentially found in open chromatin^102^. It also results in a redistribution of CTCF binding from promoters to intergenic sites, which suggests that higher-order chromatin organization may be affected by Ash2l loss. Although these findings correlate with altered H3K4 methylation, it is not understood whether the loss of H3K4me3 at promoters is necessary for the local reduced chromatin accessibility. Multiple H3K4me3 readers have been identified, which include protein complexes with histone acetyltransferase and chromatin remodeling activity^7,103–105^. Thus, the loss of H3K4me3 may cause direct effects on the accessibility of chromatin at promoters. However, it is important to note that Ash2l is an abundant protein. The analysis in HeLa cells suggests that Ash2l is considerably more abundant than all KMT2 subunits together^106^. It is possible that Ash2l possesses additional functions that do not rely on KMT2 complex activities and thus may be independent of H3K4 methylation. Future work will need to address whether so far unknown functions can be attributed to Ash2l. This will be important to clarify the contribution of H3K4 methylation to the complex phenotypes associated with Ash2l loss. Such studies will also be useful in further defining the functions of H3K4me3, in particular regarding the discussion whether this histone mark is a determinant of initiation of gene transcription or a consequence of gene transcription, for example by facilitating polymerase reinitiating and/or effects on RNA processing.

The biological responses to loss of Ash2l and H3K4 methylation are consistent with the broad effects on promoters and gene transcription. The knockout of *Ash2l* in mice in hematopoietic cells results in the accumulation of so-called LSK (lin^−^Sca1^+^Kit^+^) cells in the bone marrow. LSK cells are highly enriched in hematopoietic stem and multi-potent progenitor cells. Importantly, these cells are unable to differentiate, both in vivo and in tissue culture, and as a consequence essential mature hematopoietic cells are lacking in the animals^15^. These LSK cells accumulate over several days with strongly reduced overall H3K4me3. Thus, we suppose that the decrease in H3K4me3, most likely at promoters, results in the inability of the cells to adapt their gene expression programs for efficient differentiation. This is consistent with the above discussed functions of this histone mark as a modification that allows gene activation. While the LSK cells are arrested in G2/M, the MEF cells, both KO1 and KO2, do not respond by accumulating at a defined cell cycle stage^18^. Nevertheless, these cells stop proliferating. Phenotypically, the cells appear senescent. This is somewhat unexpected as senescence requires typically the activation of a specific gene expression program, which includes SASP (senescence-associated secretory phenotype)^107,108^. Indeed, SASP gene activation could not been observed, consistent with the broad loss of H3K4me3 at promoters. Instead, a set of downregulated genes is associated with senescence^18^. Thus, we suspect that the upregulation of SASP genes is similarly impaired as differentiation-associated genes in LSK cells. Together, these findings support the notion that H3K4me3 is important for de novo gene activation.

## Supplementary Information

Supplementary Information.

## Data Availability

Supplementary Tables S1–S5 containing ChIP-seq and ATAC-seq analyses have been deposited in Gene Expression Omnibus as SuperSeries under accession number GSE205233. This SuperSeries is composed of the following sub-series: 1. Accession number GSE205232 for ChIP-seq (H3K4me1, H3K4me3). 2. Accession number GSE205230 for ATAC-seq. 3. Accession number GSE205231 for ChIP-seq (CTCF). To review GEO accession GSE205233: Go to https://www.ncbi.nlm.nih.gov/geo/query/acc.cgi?acc=GSE205233. Three SubSeries that are linked to GSE205233: ATAC-seq: https://www.ncbi.nlm.nih.gov/geo/query/acc.cgi?acc=GSE205230; ChIP-seq (CTCF): https://www.ncbi.nlm.nih.gov/geo/query/acc.cgi?acc=GSE205231; ChIP-seq (H3K4me1, me3): https://www.ncbi.nlm.nih.gov/geo/query/acc.cgi?acc=GSE205232.
