# Supplementary material for: Loss of the Ash2l subunit of histone H3K4 methyltransferase complexes reduces chromatin accessibility at promoters
Source: Sci Rep. 2022 Dec 13;12:21506. doi: 10.1038/s41598-022-25881-0 (PMC9747801; doi:10.1038/s41598-022-25881-0)
Supplement: Supplementary file 1 — Supplementary Information. [file 41598_2022_25881_MOESM1_ESM.pdf]

Loss of the Ash2l subunit of histone H3K4 methyltransferase complexes reduces chromatin accessibility at promoters

Mirna Barsoum[1,4], Alexander T. Stenzel[1,4], Agnieszka Bochyńska[1,4], Chao-Chung Kuo[2,3,4], Alexander Tsompanidis[1], Roksaneh Sayadi-Boroujeni[1], Philip Bussmann[1], Juliane Lüscher-Firzlaff[1], Ivan G. Costa[2], and Bernhard Lüscher[1]

[1]Institute of Biochemistry and Molecular Biology, Faculty of Medicine, RWTH Aachen University, Pauwelsstrasse 30, 52074 Aachen, Germany
[2]Institute for Computational Genomics, Faculty of Medicine, RWTH Aachen University, Pauwelsstrasse 30, 52074 Aachen, Germany
[3]Interdisciplinary Center for Clinical Research (IZKF), Faculty of Medicine, RWTH Aachen University, Pauwelsstrasse 30, 52074 Aachen, Germany
[4]Contributed equally
Correspondence to B.L. (luescher@rwth-aachen.de)

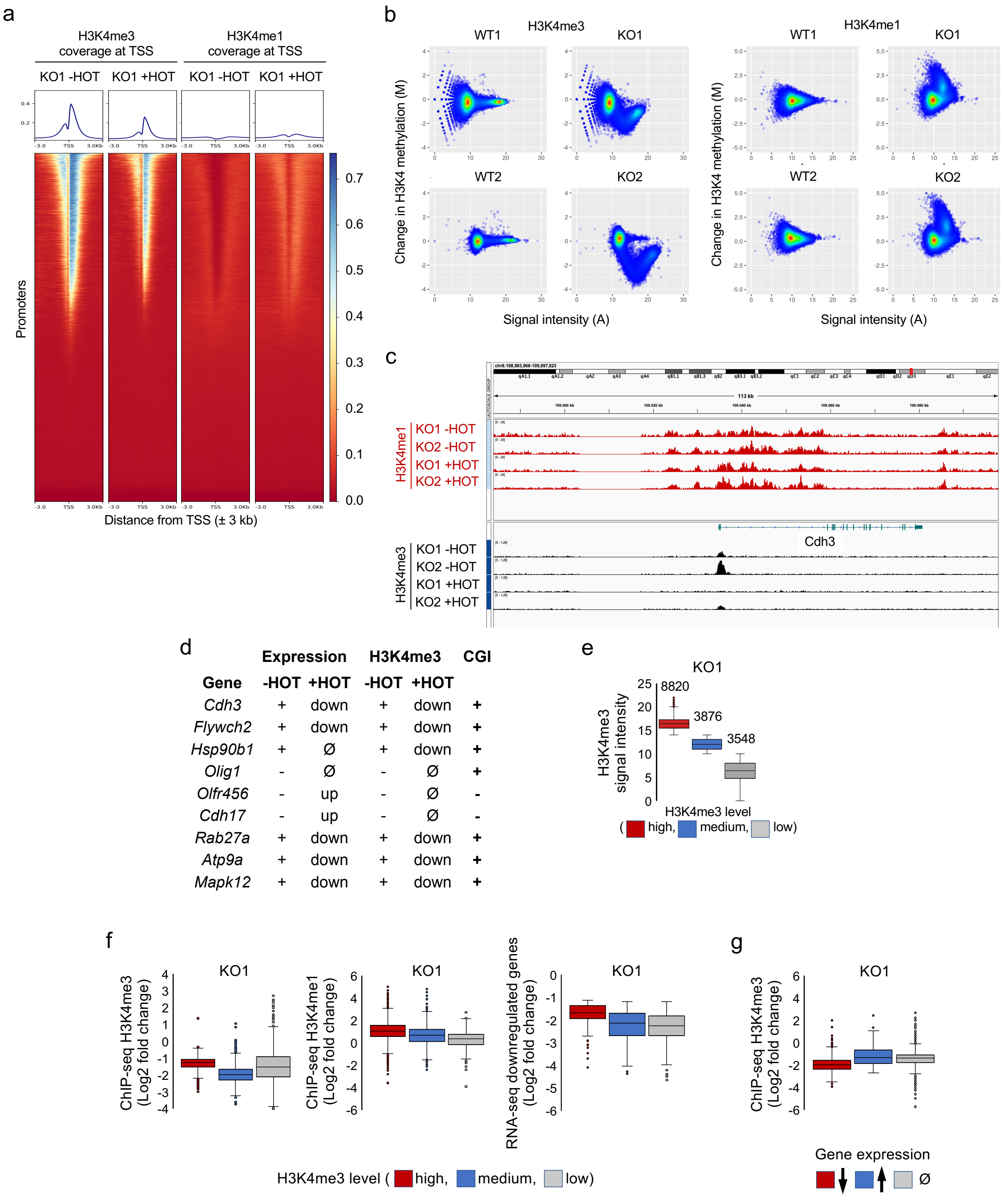

Supplementary Figure S1

Related to Figure 1.

a. Heatmaps generated using DeepTools showing H3K4me3 and me1 normalized signals (normalized using counts per Million (CPM)) obtained from KO1 cells in the presence and absence of Ash2l (±HOT) at all annotated transcripts in mm9.

b. MA plots (A = 0.5(log2(KO+HOT) + log2(KO-HOT)); M = log2(KO+HOT) – log2(KO-HOT)) of H3K4me3 and me1 ChIP-seq data.

c. Displayed are IGV browser tracks from H3K4me3 and me1 ChIP-seq data of KO1 and KO2 cells treated ±HOT for 5 days. The BigWig tracks were normalized as in panel a.

d. Summary of the expression and H3K4me3 promoter modification of the indicated genes in response to Ash2l depletion.
e. Promoters were classified according to their H3K4me3 levels. Three categories were generated, the numbers of promoters in each category are indicated.
f. Promoters (±3000 bp of TSS) with high, medium and low H3K4me3 signals were compared regarding alterations in H3K4me1 and H3K4me3 and changes in expression of the corresponding genes.
g. Genes that are up- or downregulated or did not change in expression were compared to changes in H3K4me3 at their promoters.

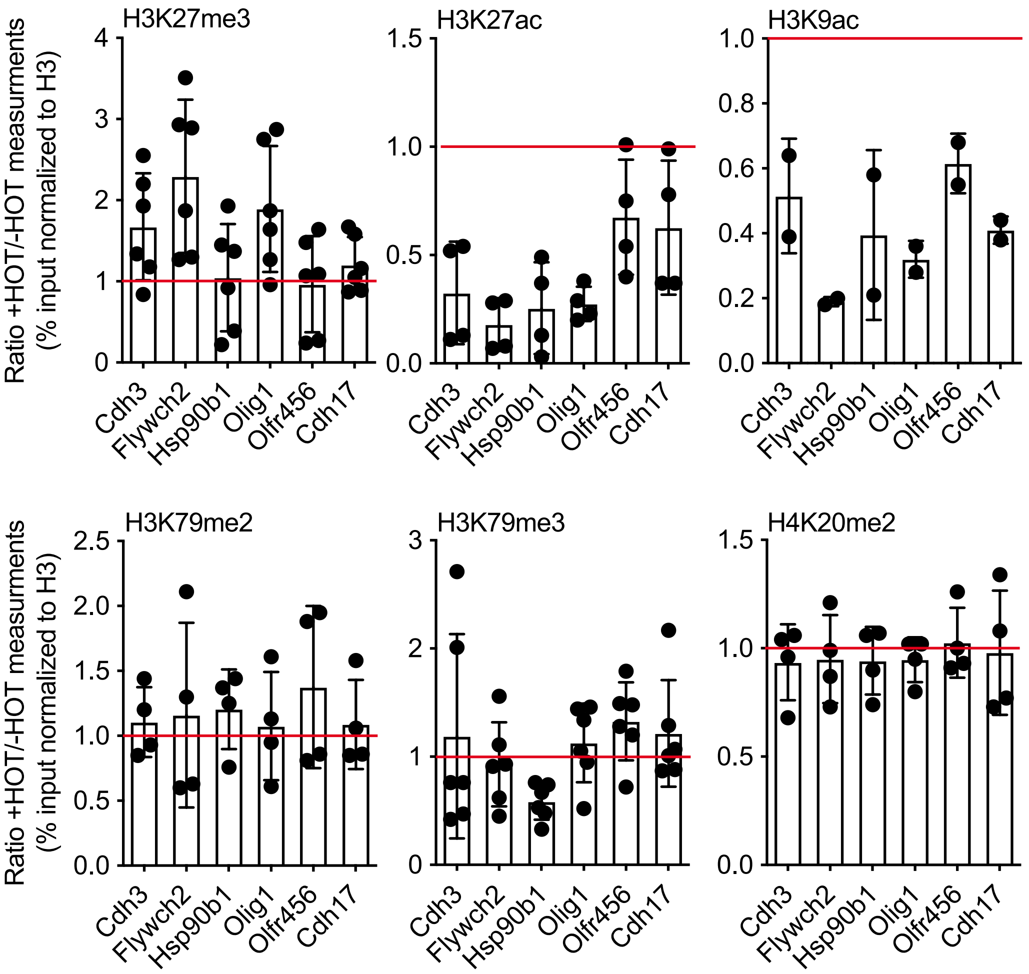

Supplementary Figure S2

Related to Figure 2.

The indicated histone marks were analyzed with selective antibodies using ChIP-qPCR. The data were normalized to the histone H3 signal obtained in the same experiments. Mean values ± SD (n = 2-6).

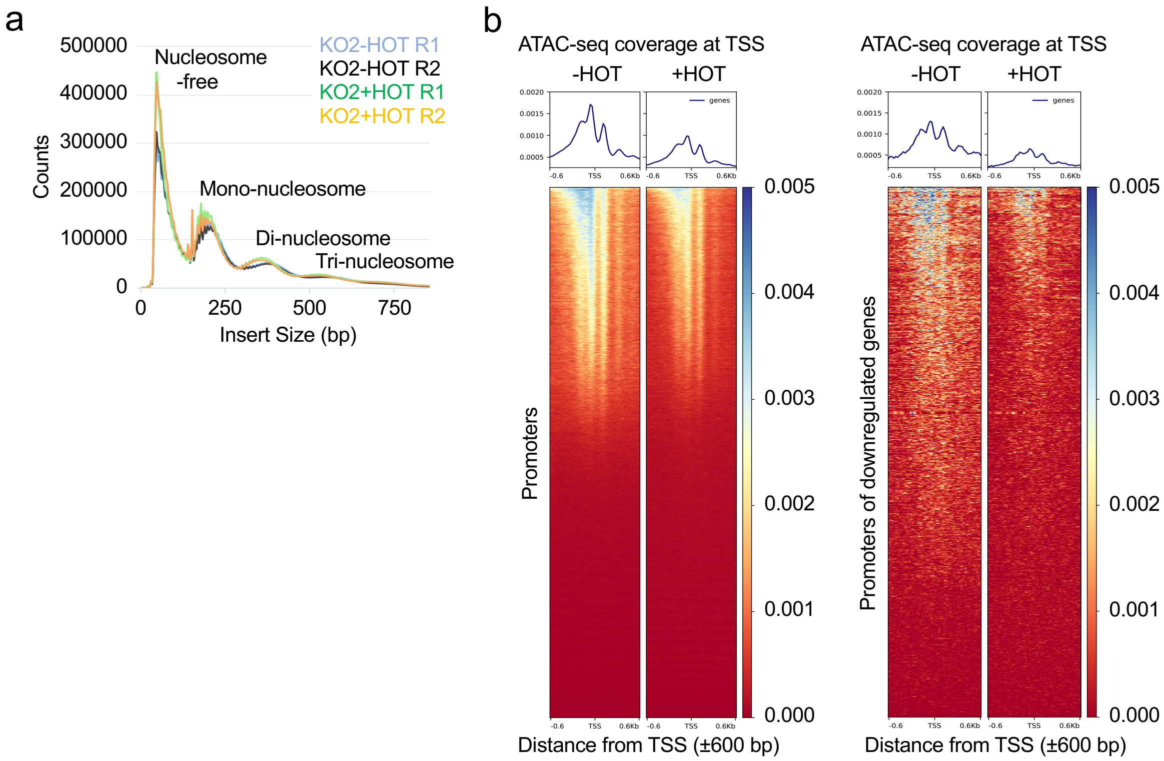

Supplementary Figure S3

Related to Figure 3.

a. ATAC-seq was performed in KO2 cells (2 replicates), which were treated ±HOT for 7 days. Insert size distribution plot generated using multiQC showing that appropriate data was collected. Indicated are peaks at nucleosome-free, mono-nucleosome, di-nucleosomes and tri-nucleosomes.

b. Heatmaps generated using DeepTools as in panel C using a window of ±600 bp of TSS. Promoters of all annotated transcripts in mm9 are shown on the left, promoters of the downregulated genes are displayed on the right.

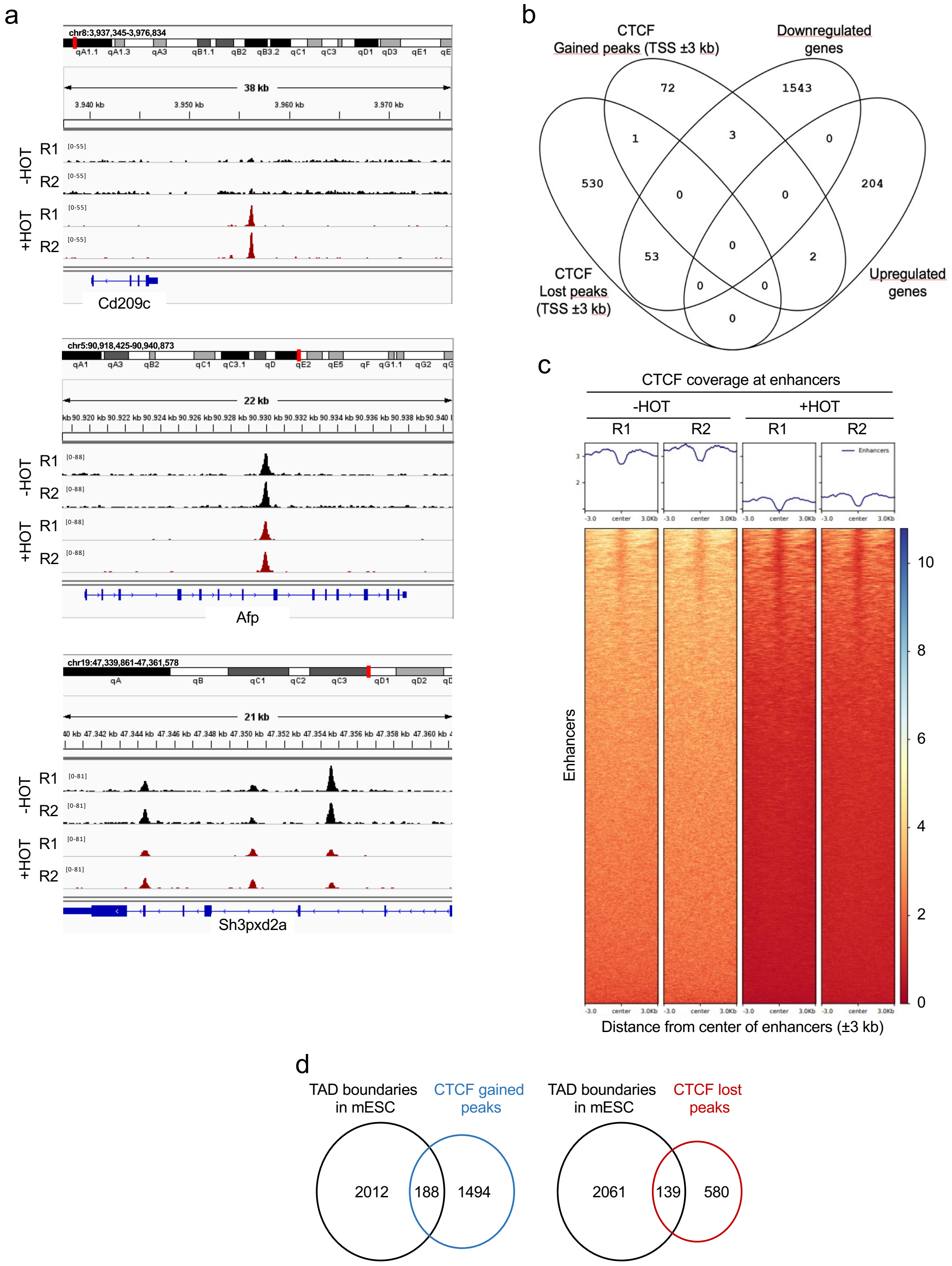

Supplementary Figure S4

Related to Figure 4.

a. Integrated Genome Viewer (IGV) of three loci are shown: gained binding (Chr8:3,955,970-3,956,485) (top panel), unaffected binding (Chr5:90,929,711-90,930,343) (middle panel), and lost binding (Chr19:47,354,359-47,354,913) (bottom panel), The BigWig tracks were normalized using the scale factors obtained by Deseq2.

b. Up- and downregulated genes obtained from RNA-seq (Bochynska et al. NAR submitted) were compared to gained and lost binding sites from CTCF ChIP-seq.
c. Heatmaps generated using DeepTools showing the normalized signal of ChIP-seq (CTCF; 2 replicates; normalized using the scale factors obtained by Deseq2) in the presence and absence of Ash2l (±HOT) centered at annotated enhancers (±3000 bp) in MEF cells (E13.5).
d. Overlap of gained and lost CTCF binding sites defined in ChIP-seq upon loss of Ash2l with known TAD boundaries in mESCs (±20 kb) [1].

REFERENCE

1. Dixon, J. R. *et al.* Topological domains in mammalian genomes identified by analysis of chromatin interactions. *Nature* **485**, 376-380, doi:10.1038/nature11082 (2012).
